# Solitary Dermal Cylindroma in an Uncommon Site: A Rare Case Report

**DOI:** 10.7759/cureus.32614

**Published:** 2022-12-16

**Authors:** Lakshmi Priya V., Thanka Johnson, Srismitha S., Shobana Balakrishnan

**Affiliations:** 1 Department of Pathology, Sree Balaji Medical College and Hospital, Chennai, IND

**Keywords:** pas, left side chest wall, benign, solitary, dermal cylindroma

## Abstract

Cylindroma, a benign skin appendage tumor, mostly occurs in the head and neck region. They present as pink to purple, smooth-surfaced, solitary or multiple nodular lesions, mostly affecting middle-aged and elderly women. A clinical diagnosis of cylindroma is unusual but must be considered clinically. We report an unusual case of solitary benign dermal cylindroma that occurred in an uncommon site in a 54-year-old female. She came with complaints of painful swelling on the left side of her chest wall for six months. Histopathological examination of the mass revealed a well-defined tumor composed of nests and islands of a dual population of tumor cells surrounded by pink basement membrane-like material. These nests of cells are arranged in a "jigsaw" puzzle fashion, which is typical of cutaneous cylindroma. The thick pink material surrounding the epithelial cell islands is periodic acid Schiff stain (PAS) positive.

## Introduction

Cylindroma is a benign skin appendage neoplasm. It is a rare, slow-growing tumor, accounting for 0.7% of all adnexal neoplasms [1]. It mostly occurs in elderly females. The preferred site of the cylindroma is often in the scalp, face, and neck region. Despite the development of multiple theories about its origin, pathogenesis is still not well established. The term cylindroma was initially coined by Billroth during the description of adenoid cystic carcinoma (ADCC) in the year 1959 [2]. When these cells are viewed at the cross-section, the morphology of cells will be round resembling cylinders. Hence termed as cylindroma [2,3].

They present either as solitary or multiple tumors [4]. Solitary presentations are usually sporadic and occur in the elderly age, often having expressions of V-Myb avian myeloblastosis viral oncogene homolog and nuclear factor I/B (MYB-NFIB) fusion transcripts. This fusion transcript acts as a trigger for sporadic dermal cylindroma to occur. Multiple cutaneous cylindromas are mostly familial inherited and manifest often at an earlier age. A mutation in the tumor suppressor CYLD gene is implicated as the cause for inherited cylindromatosis syndromes. Three inherited cylindromatosis syndromes have been identified so far. Of which, Brooke-Spiegler syndrome is the most well-known. The other two inherited cylindromatosis syndromes are familial cylindromatosis and multiple familial trichoepitheliomas. So far, 100 and more mutations in the CYLD gene have been identified that are responsible for varied phenotypic expression in affected members of the same family. They are generally benign tumors but can undergo malignant transformations, mostly in patients with Brooke-Spiegler syndrome [2].

Here, we report an unusual case of solitary benign dermal cylindroma in the left side chest wall of a 54-year-old female.

## Case presentation

**Figure 1:**
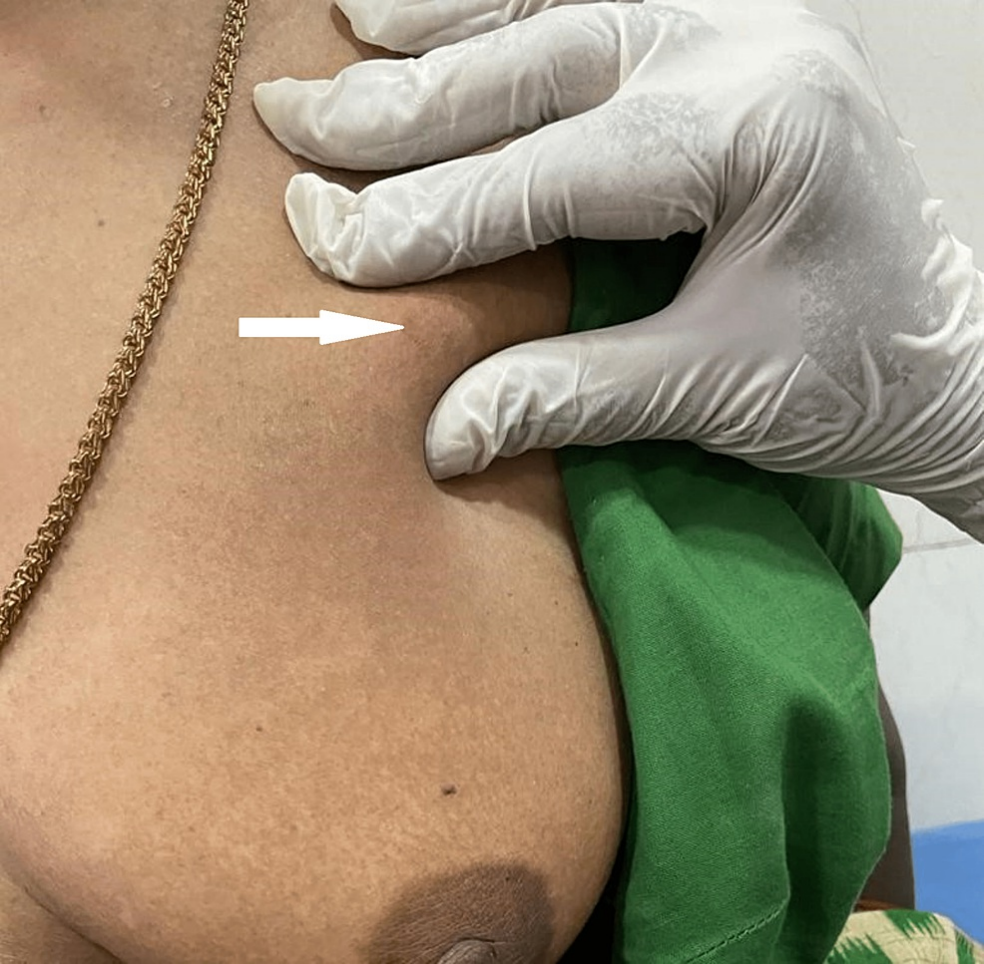
Nodular smooth-surfaced swelling on the left side of the chest wall.

A 54-year-old woman presented with a painful swelling on the left side of her chest wall over a period of six months. The past surgical, medical, and family histories are not remarkable. On local examination, solitary nodular smooth-surfaced swelling of size 1 × 1 cm, which was firm, non-tender, and mobile (Figure 1). The clinical diagnosis was neurofibroma. The patient underwent surgical excision. The excised specimen was sent for histopathological examination.

**Figure 2:**
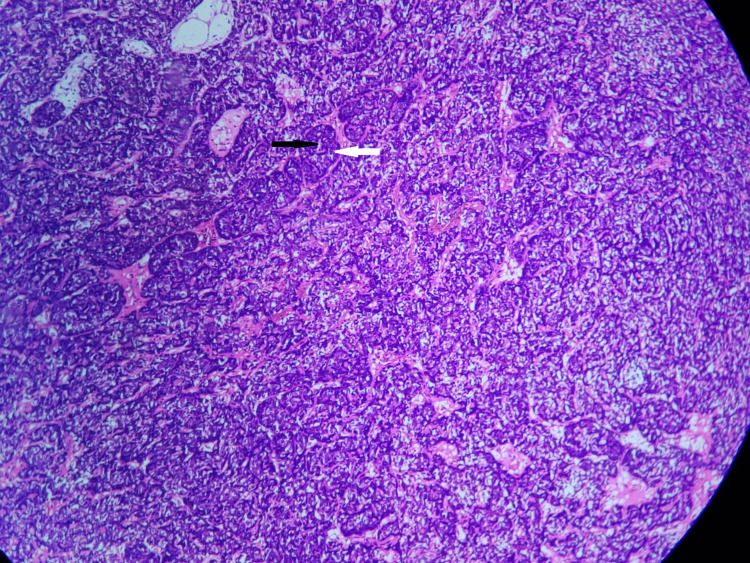
Low power 10×: hematoxylin and eosin stain-solid and irregular cell nests arranged in a jigsaw puzzle fashion. Black arrow represents irregular cell nests. White arrow represents the eosinophilic material surrounding these epithelial cell nests, which imparts a jigsaw puzzle fashion to this tumor.

**Figure 3:**
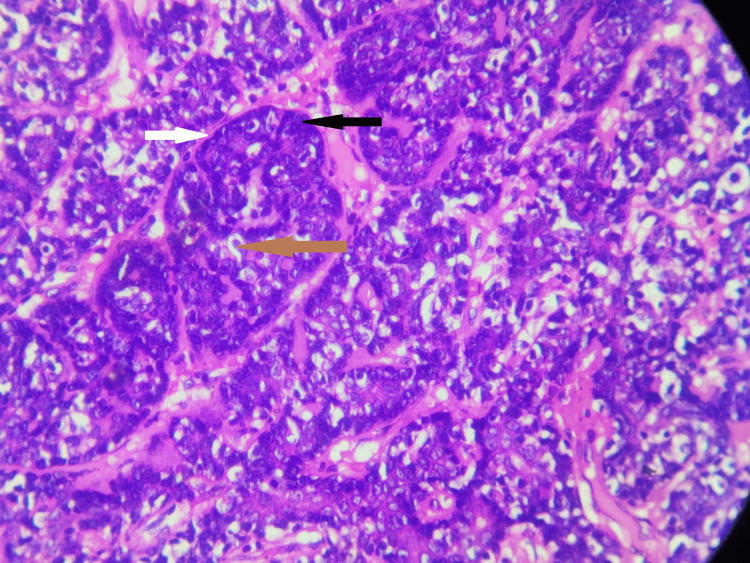
High power 40×: hematoxylin and eosin stain-solid and irregular cell nests composed of a dual cell population, which are small palisading basal-like cells having scant cytoplasm and a dark nucleus in the periphery and large pale cells located in the center having moderately pale pink cytoplasm and vesicular nuclei. The pink eosinophilic material is seen surrounding these tumor cell nests. Black arrow represents small, palisading basal-like cells having scant cytoplasm and a dark nucleus in the periphery. Brown arrow represents large pale cells located in the center and having moderately pale pink cytoplasm and a vesicular nucleus. White arrow represents the eosinophilic basement membrane-like material is seen surrounding these tumor cell nests.

**Figure 4:**
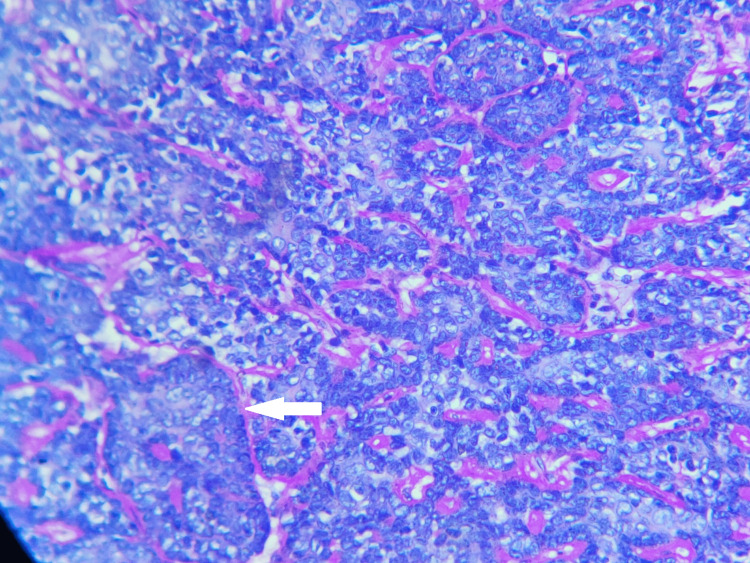
Periodic acid Schiff stain-pink eosinophilic material is seen surrounding these tumor cell nests are periodic acid Schiff stain positive. White arrow represents eosinophilic basement membrane-like material are periodic acid Schiff stain positive.

The specimen was fixed in 10% buffered formalin. Gross examination revealed a skin-covered, gray-white firm mass of size 1 × 1 × 0.7 cm. Microscopically, it revealed skin with subcutis and a well-demarcated un-encapsulated tumor within the dermis, consisting of biphasic proliferation of basaloid and ductal or secretory cells reminiscent of “jigsaw” puzzle fashion and has a prominent pink material surrounding these epithelial nests at low power. The dual cell proliferations are small palisading basal-like cells with scant cytoplasm and a dark nucleus in the periphery and large pale cells located in the center with moderately pale pink cytoplasm and vesicular nuclei (Figures 2, 3). No necrosis and nuclear atypia were noted. Mitosis was 0-1 per 10 high-power fields (HPFs). Surgical margins are negative. The prominent eosinophilic material surrounding these tumor nests is periodic acid Schiff stain (PAS) positive (Figure 4). Histopathologically, it was diagnosed as a benign dermal cylindroma in the left chest wall.

## Discussion

Dermal cylindromas are usually found in the scalp and face [5], but they also occur in the trunk and extremities [2]. Clinically, sporadic cylindromas usually appear as solitary, smooth-surfaced, dome-shaped nodules that are usually small-sized [6]. They are nine times more frequent in females than males [7]. Multiple lesions are seen in familial inherited cylindromatosis, usually autosomal dominant. When multiple cylindromatosis occurs in the scalp region, they resemble a hat or a turban configuration, hence previously called “turban tumor’’ [2]. The presence of spiradenomas and trichoepitheliomas, in addition to cylindromas, forms the Brooke-Spiegler syndrome [7]. Gene testing for the CYLD gene located at 16q12-q13 can be done for this syndrome when it is suspected [8].

Conventionally, cylindromas are classified under tumors of apocrine origin because more differentiated cells morphologically resemble secretory cells, which argues in favor of apocrine origin. The ultrastructure and immunohistochemistry studies state that it could be of eccrine origin [9,10]. According to Massoumi et al. [11], hair follicle-covered areas are the potential sites, which are of follicular epithelial lineage, and they don't appear at the palm and sole of the hands and feet, respectively. This statement supports that these tumors are originating from hair follicles [11,12]. However, the histogenesis of cylindromas still remains uncertain despite the development of various immunohistological, histochemical, and ultrastructure studies.

The tendency of malignancy is higher in multiple cylindromas than in the solitary type. Only less than 50 malignant cylindroma cases are reported [10,13]. Clinical signs that suggest malignant transformation are sudden and rapid tumor growth, necrosis, ulceration, blue-to-pinkish discoloration, and hemorrhage [7].

Histopathologically, it has irregular islands and nested proliferation of tumor cells arranged in a ‘‘jigsaw" puzzle fashion. These cells have an eosinophilic pink material surrounding these epithelial cell nests [12]. These tumors typically have a biphasic cell population: peripherally located palisading cells that have a small dark nucleus, representing undifferentiated cells, and centralized differentiated cells having a medium- to large-sized pale nucleus, morphologically representing ductal or secretory cells [14]. Differential diagnoses of dermal cylindromas are spiradenoma and adenoid cystic carcinoma-solid variants [3,8,15]. Sometimes they may occur with spiradenoma and are termed as cylindroma-spiradenoma. Spiradenoma can be differentiated by the absence of pink basement membrane material that tends to be more vascular and shows tubular differentiation with lymphocyte infiltration [15]. The adenoid cystic carcinoma-solid variant is differentiated from cylindromas by its infiltrating pattern, atypical nuclear features, and mitosis [3,8].

Complete excision by surgery is the treatment of choice for cases of dermal cylindromas. It has a 40% recurrence rate with a rare possibility of malignant transformation [7]. Therefore, close follow-up is required. In cases of multiple cylindromatosis, in addition to resection, treatment with a CO_2_ laser is needed to avoid recurrences [7].

## Conclusions

Solitary dermal cylindroma is a rare tumor to be diagnosed in clinical practice, especially when it occurs in an uncommon site. Histopathological examination is required for a definitive diagnosis as the clinical presentation is often indistinct. Complete excision of the tumor is the treatment of choice. Because of the possibility of recurrence, close follow-up with the patient is required. In cases of inherited cylindromatosis, gene testing for the CYLD mutation will be of great importance.
